# Supplementary material for: Phosphorylated Peptide Derived from the Myosin Phosphatase Target Subunit Is a Novel Inhibitor of Protein Phosphatase-1
Source: Int J Mol Sci. 2023 Mar 1;24(5):4789. doi: 10.3390/ijms24054789 (PMC10003451; doi:10.3390/ijms24054789)
Supplement: Supplementary file 1 [file ijms-24-04789-s001.zip › ijms-2201369-supplementary.pdf]

International Journal of Molecular Sciences

Supplementary information

Phosphorylated peptide derived from the myosin phosphatase target subunit is a novel inhibitor of protein phosphatase-1

Zoltán Kónya[1], István Tamás[1], Bálint Bécsi[1], Beáta Lontay[1], Mária Raics[2], István Timári[3], Katalin E. Kövér[2,4], Ferenc Erdődi[1,*]

1 Department of Medical Chemistry, Faculty of Medicine, University of Debrecen, H-4032 Debrecen, Hungary
2 Department of Inorganic and Analytical Chemistry, Faculty of Natural Science and Technology, University of Debrecen, H-4032 Debrecen, Hungary
3 Department of Organic Chemistry, Faculty of Natural Science and Technology, University of Debrecen, H-4032 Debrecen, Hungary
4 MTA-DE Molecular Recognition and Interaction Research Group, University of Debrecen, H-4032 Debrecen, Hungary
* Correspondence: Dr. Ferenc Erdődi; Tel.: +36-52-412345; Fax: +36-52-412566; erdodi@med.unideb.hu;

SUPPLEMENTARY INFORMATION

Figure S1. Saturation Transfer Difference (STD) ^{1}H-NMR spectra of P-MYPT1$^{690\text{-}701}$ recorded in the absence and in the presence of recombinant PP1c.

Figure S2. Original dot blots to the data of Fig. 4B

Table S1 HADDOCK scores and energies for the poses of docked P-Ser696-MYPT1$^{690\text{-}701}$ and P-Thr696-MYPT1$^{690\text{-}701}$ peptides on the surface of PP1c

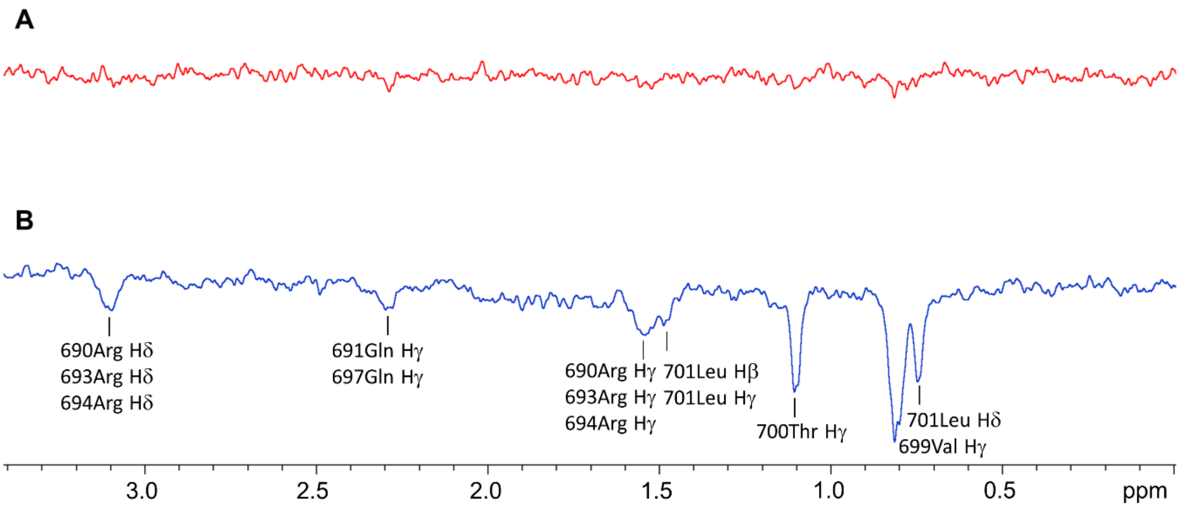

Figure S1. Saturation Transfer Difference (STD) 1H-NMR spectra of P-Thr696-MYPT1$^{690\text{-}701}$ (1.3 mM) recorded in the absence (**A**) and in the presence of 13.3 μM recombinant PP1c (**B**). The lack of signals in the control STD spectrum (**A**) confirms that selective irradiation applied at -650 Hz (-1.3 ppm) has no partial saturation effect on the P-Thr696-MYPT1$^{690\text{-}701}$ signal intensities. Thus, the STD signals in spectrum (**B**) are solely due to saturation transferred from the protein upon binding interaction. All relevant experimental parameters are detailed in section 4.6.

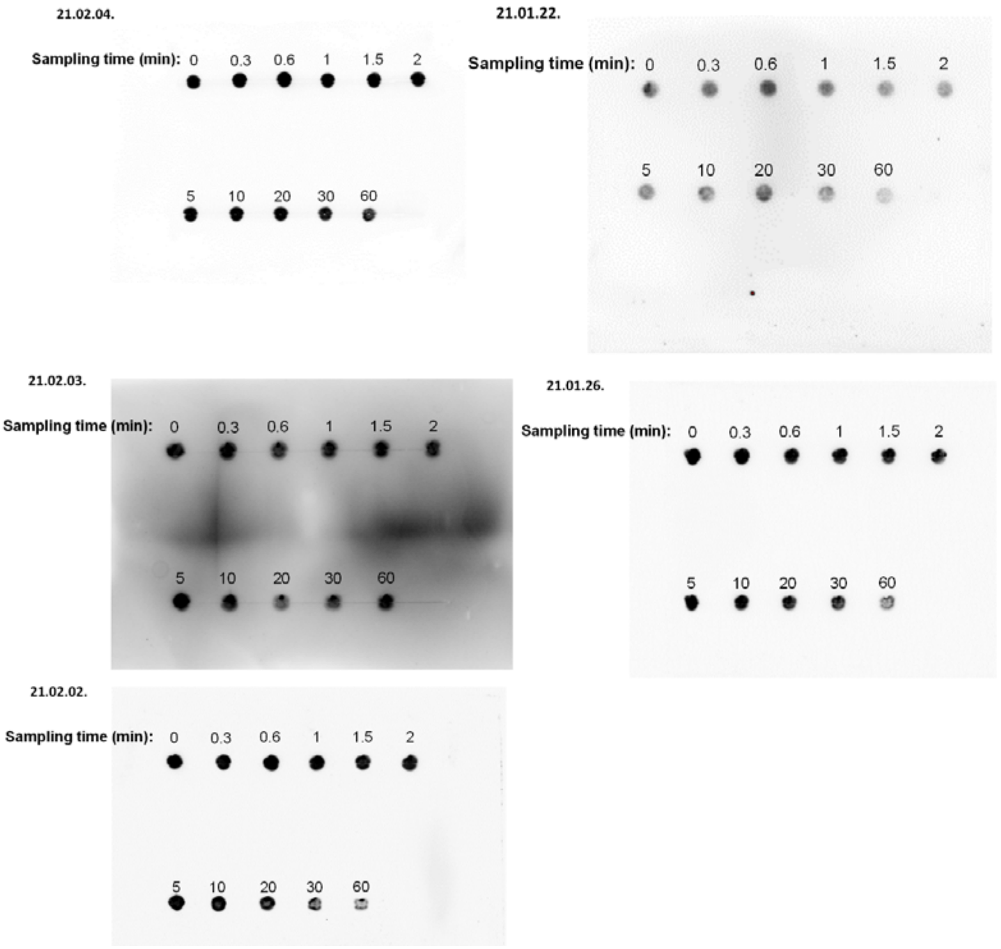

Figure S2. Original dot blots to the data of Fig. 4B

Table S1 HADDOCK scores and energies for the poses of docked P-Ser696-MYPT1$^{690-701}$ and P-Thr696-MYPT1$^{690-701}$ peptides on the surface of PP1c

	**P-Ser696-MYPT1$^{690-701}$**	**P-Thr696-MYPT1$^{690-701}$**
HADDOCK score	-175.6 +/- 14.2	-165.6 +/- 18.1
RMSD from the overall lowest-energy structure	1.6 +/- 1.6	3.3 +/- 0.3
Van der Waals energy	-7.0 +/- 13.8	-26.8 +/- 12.4
Electrostatic energy	-950.0 +/- 99.3	-793.3 +/- 79.3
Desolvation energy	12.0 +/- 1.9	8.3 +/- 3.6
Restraints violation energy	93.4 +/- 49.44	114.6 +/- 50.32
Buried Surface Area	1725.6 +/- 104.9	1993.9 +/- 159.2
Z-Score	-0.5	-0.6
